# Supplementary material for: Tip60-mediated lipin 1 acetylation and ER translocation determine triacylglycerol synthesis rate
Source: Nat Commun. 2018 May 15;9:1916. doi: 10.1038/s41467-018-04363-w (PMC5953937; doi:10.1038/s41467-018-04363-w)
Supplement: Supplementary file 1 — Supplementary Information [file 41467_2018_4363_MOESM1_ESM.pdf]

Supplementary Information

# Tip60-mediated lipin 1 acetylation and ER translocation determine triacylglycerol synthesis rate

Li *et al*.

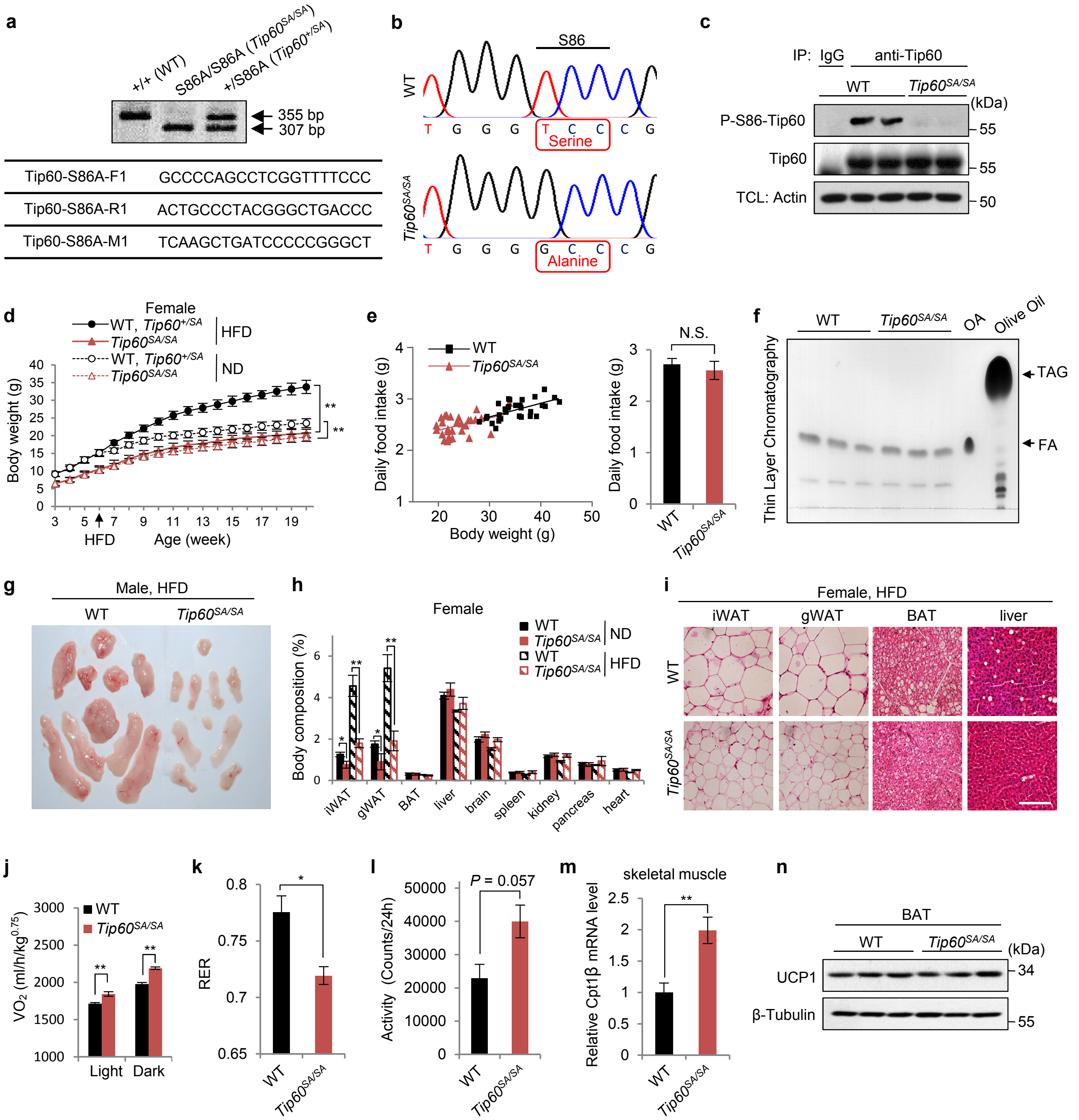

a
+/+ (WT)
S86A/S86A (Tip60SA/SA)
+/S86A (Tip60+/SA)
355 bp
307 bp
Tip60-S86A-F1 GCCCCAGCCTCGGTTTTCCC
Tip60-S86A-R1 ACTGCCCTACGGGCTGACCC
Tip60-S86A-M1 TCAAGCTGATCCCCCGGGCT
b
S86
WT
Serine
Tip60SA/SA
Alanine
c
IP: IgG anti-Tip60
WT Tip60SA/SA
(kDa)
P-S86-Tip60
Tip60
TCL: Actin
d
Female
WT, Tip60+/SA
Tip60SA/SA
HFD
ND
Body weight (g)
Age (week)
e
WT
Tip60SA/SA
Daily food intake (g)
Body weight (g)
N.S.
f
WT Tip60SA/SA OA Olive Oil
Thin Layer Chromatography
TAG
FA
g
Male, HFD
WT Tip60SA/SA
h
Female
Body composition (%)
iWAT gWAT BAT liver brain spleen kidney pancreas heart
i
Female, HFD
iWAT gWAT BAT liver
j
VO2 (ml/h/kg0.75)
Light Dark
k
RER
l
Activity (Counts/24h)
P = 0.057
m
skeletal muscle
Relative Cpt1β mRNA level
n
BAT
UCP1
β-Tubulin

**Supplementary Fig. 1** *Tip60$^{SA/SA}$* mice demonstrate lean phenotypes and are resistant to obesity-associated metabolic disorders. (**a**) PCR analysis of wild-type (WT), heterozygous (*Tip60$^{+/SA}$*) or *Tip60$^{SA/SA}$* knock-in mice and the primers used for genotyping. (**b**) Nucleotide sequencing results of genomic DNA from WT and *Tip60$^{SA/SA}$* mice. (**c**) Equal amounts of cell lysates from WT or *Tip60$^{SA/SA}$* mouse embryonic fibroblasts (MEFs) were immunoprecipitated by antibody to Tip60 and analyzed by immunoblotting as indicated. (**d**) Body weights of female WT, *Tip60$^{+/SA}$* and *Tip60$^{SA/SA}$* mice fed a ND *(n* = 16 per group) or HFD starting at 6-week age (*n* = 20 per group). (**e**) Daily food intake of HFD-fed WT and *Tip60$^{SA/SA}$* mice (male, *n* = 25 per group). Food intake and body weight were collected for 4 consecutive weeks. Squares and triangles correspond to single individuals. Bar graph (right) shows the adjusted means based on a normalized mouse weight of 27.9 g determined by using ANCOVA. (**f**) No evidence of increased fecal fat content in *Tip60$^{SA/SA}$* mice. Total lipids were extracted from fecal collected from WT and *Tip60$^{SA/SA}$* mice fed HFD for one week and separated by thin layer chromatography (TLC). OA, oleic acid; TAG, triacylglycerol; FA, fatty acid. (**g**) Representative photographs of different positions of white adipose tissue (WAT) explants isolated from 18-week old WT and *Tip60$^{SA/SA}$* HFD male mice. (**h**) Percentages of the weights of different tissues from 18-week old WT and *Tip60$^{SA/SA}$* ND or HFD female mice as compared to their corresponding body weights (*n* = 12 per group). iWAT, inguinal WAT; gWAT, gonadal WAT; BAT, brown adipose tissue. (**i**) Representative sections of iWAT, gWAT, BAT and liver from HFD 18-week old female mice. Scale bar, 50 μm. (**j-m**) $O_2$ consumption ($VO_2$) (**j**), respiratory exchange ratio (RER) (**k**), physical activity (**l**), and gene expression level of *Cpt1β* (**m**) of WT and *Tip60$^{SA/SA}$* mice fed HFD for one week (male, *n* = 6 per group), before the mice diverge too much in body weight. For $VO_2$ data were collected for 3 consecutive days, expressed as adjusted means based on body weight to the power 0.75. (**n**) Similar expression level of Ucp1 in brown adipose tissue (BAT) of WT and *Tip60$^{SA/SA}$* mice fed HFD for one week (male, *n* = 6 per group). Error bars denote SEM. Statistical analysis was performed by ANOVA followed by Tukey in (**d**, **h**) and (**j**), or by two-tailed unpaired Student's *t*-test in (**k-m**) (**P* < 0.05; ***P* < 0.01; N.S., not significant). Uncropped blots can be found in Supplementary Fig. 6.

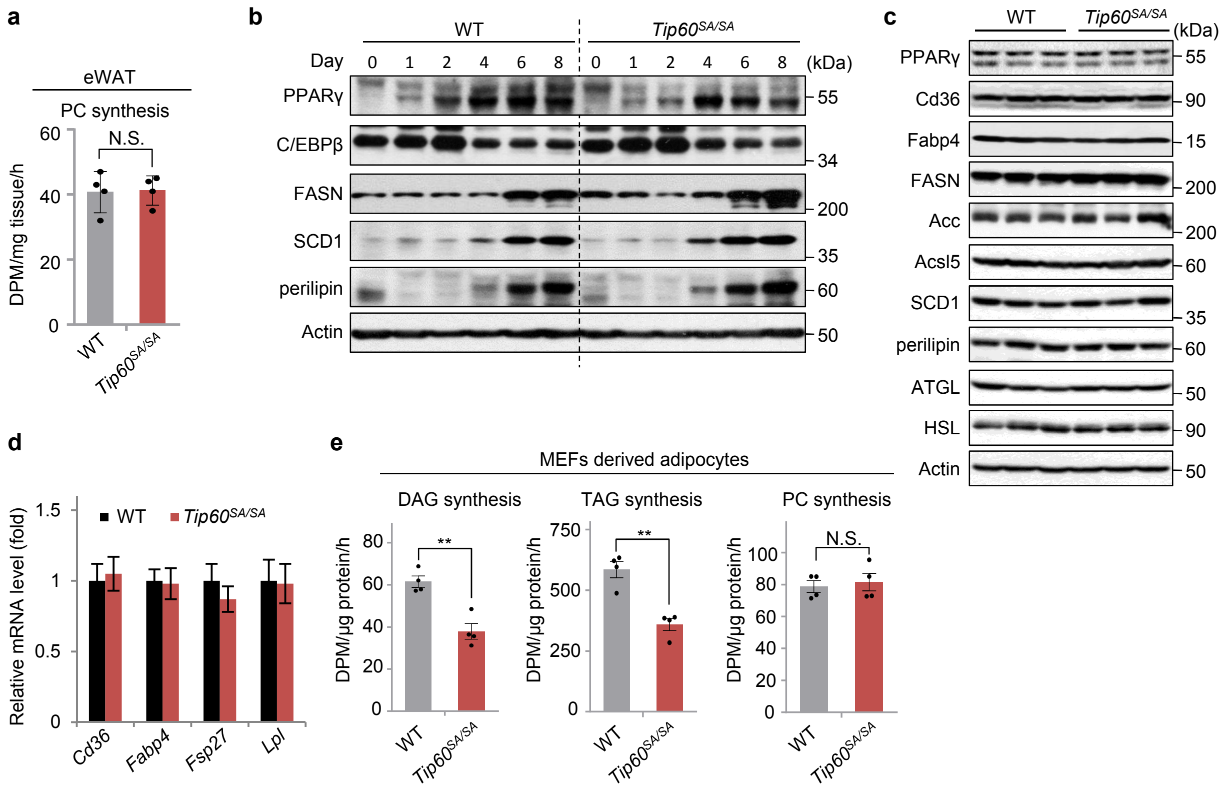


**Supplementary Fig. 2** Roles of Tip60 and its Ser[86] phosphorylation in adipogenesis and lipid synthesis. (**a**) Rates of PC synthesis from $^{3}$H-OA in eWAT explants isolated from 6-week old WT and *Tip60$^{SA/SA}$* mice (*n* = 4 individuals per group). (**b**) Cell lysates of WT and *Tip60$^{SA/SA}$* MEFs after 0-8 days of adipogenic differentiation induction were analysed by immunoblotting. (**c**) Cell lysates of WT and *Tip60$^{SA/SA}$* stromal vascular fibroblasts (SVFs) after 8 days of adipogenic differentiation induction were analysed by immunoblotting. (**d**) RT-PCR analysis of WT and *Tip60$^{SA/SA}$* SVFs after 8 days of differentiation induction (right, *n* = 8 mice for each group). (**e**) Rates of DAG, TAG and PC synthesis in WT and *Tip60$^{SA/SA}$* MEFs after 8 days of differentiation (*n* = 4 experiments). TAG, triacylglycerol, DAG, diacylglycerol, PC, phosphatidylcholine. Error bars denote SEM. Statistical analysis was performed by two-tailed unpaired Student's *t*-test in (**a**) and (**e**) (**$P < 0.01$; N.S., not significant). Uncropped blots can be found in Supplementary Fig. 6.

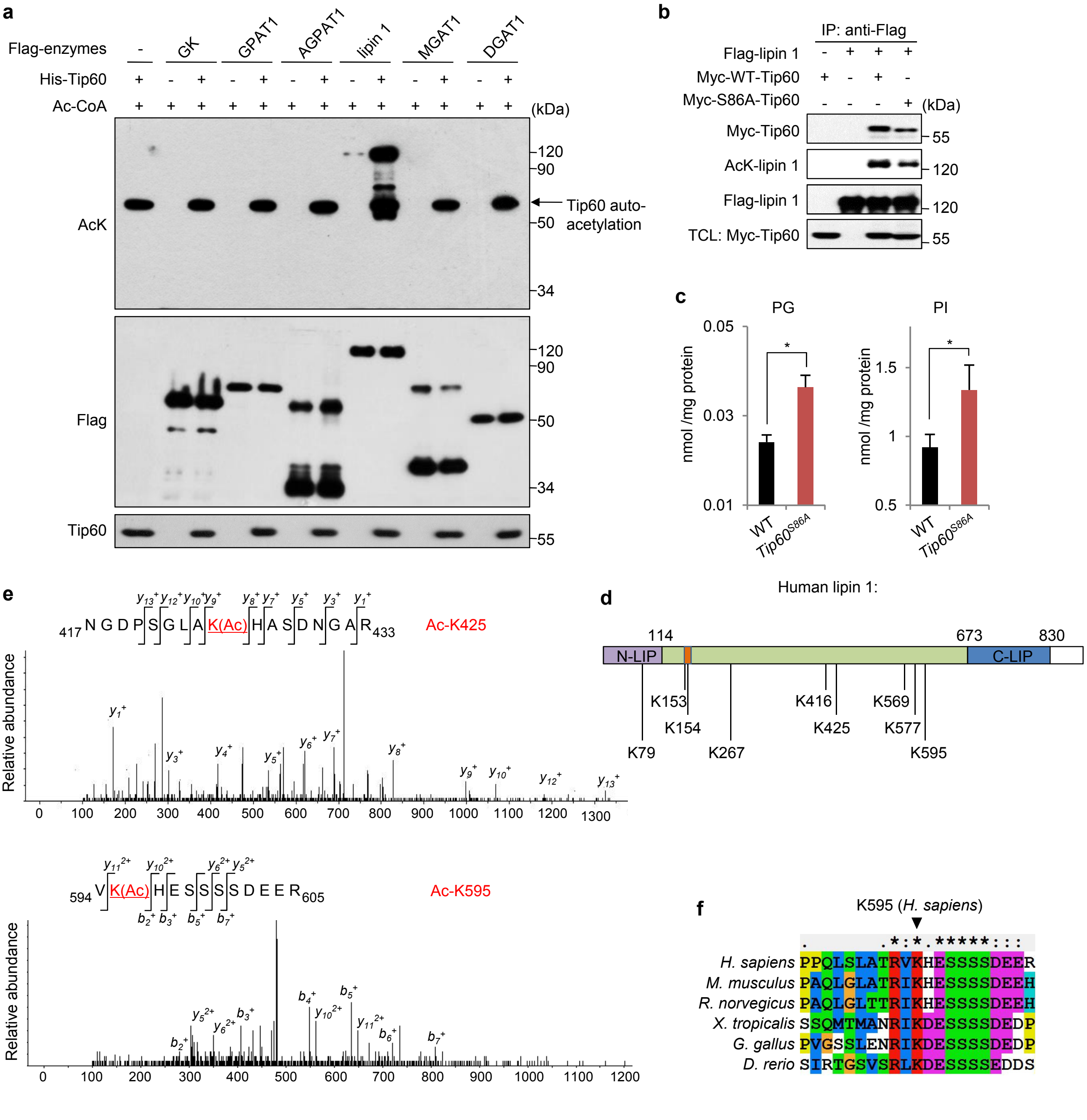

a
Flag-enzymes
GK
GPAT1
AGPAT1
lipin 1
MGAT1
DGAT1
His-Tip60
Ac-CoA
(kDa)
AcK
Tip60 auto-acetylation
Flag
Tip60
120
90
50
34
55
b
IP: anti-Flag
Flag-lipin 1
Myc-WT-Tip60
Myc-S86A-Tip60
Myc-Tip60
AcK-lipin 1
Flag-lipin 1
TCL: Myc-Tip60
c
PG
PI
nmol /mg protein
WT
Tip60S86A
d
Human lipin 1:
114
673
830
N-LIP
C-LIP
K79
K153
K154
K267
K416
K425
K569
K577
K595
e
417 N G D P S G L A K(Ac) H A S D N G A R 433
Ac-K425
Relative abundance
594 V K(Ac) H E S S S S D E E R 605
Ac-K595
f
K595 (H. sapiens)
H. sapiens PPQLSLATRVKHESSSSDEER
M. musculus PAQLGLATRIKHESSSSDEEH
R. norvegicus PAQLGLTTRIKHESSSSDEEH
X. tropicalis SSQMTMANRIKDESSSSDEDP
G. gallus PVGSSLENRIKDESSSSDEDP
D. rerio SIRTGSVSRLKDESSSSEDDS

**Supplementary Fig. 3** Identification of Lys$^{425}$ and Lys$^{595}$ as amino acid sites on lipin 1 acetylated by Tip60. (**a**) Acetylation of lipin 1 by Tip60. Tip60 specifically acetylates lipin 1 but not other enzymes in the TAG synthesis pathway. Immunoprecipitated Flag-tagged enzymes in the TAG biosynthesis pathway were incubated with or without bacterially expressed His-Tip60 in the presence of Ac-CoA and immunoblotted as indicated. (**b**) S86A-Tip60 mutant demonstrates attenuated binding affinity with lipin 1. HEK293T cells were transfected with Flag tagged lipin 1 and Myc tagged WT or S86A mutant of Tip60, immunoprecipitated with antibody to Flag and immunoblotted as indicated. (**c**) Total levels of phosphatidylglycerol (PG) and phosphatidylinositol (PI) normalized to cellular protein in eWAT from 6-week old WT and *Tip60*$^{SA/SA}$ mice ($n$ = 3 individuals for each group). (**d**) Mass spectrometric analysis for acetylation sites on lipin 1. N-LIP, N-terminus lipin homology domain; C-NLP, C-terminus lipin homology domain. (**e**) Identification of K425 and K595 acetylation on lipin 1 by liquid chromatography-mass spectrometry (LC-MS/MS) analysis. The MS/MS spectrum of the acetylated peptide NGDPSGLAK(Ac)HASDNGAR (top) and VK(Ac)HESSSSDEER (bottom) are shown. The labeled peaks show the masses of $y$ or $b$ ions of the acetylated peptides. (**f**) Sequence alignment of the residues flanking Lys$^{595}$ across different species. Arrow heads point to Lys$^{595}$ residues corresponding to human lipin 1. Error bars denote SEM. Statistical analysis was performed by two-tailed unpaired Student's $t$-test in (**b**) ($^*P < 0.05$). Uncropped blots can be found in Supplementary Fig. 6.

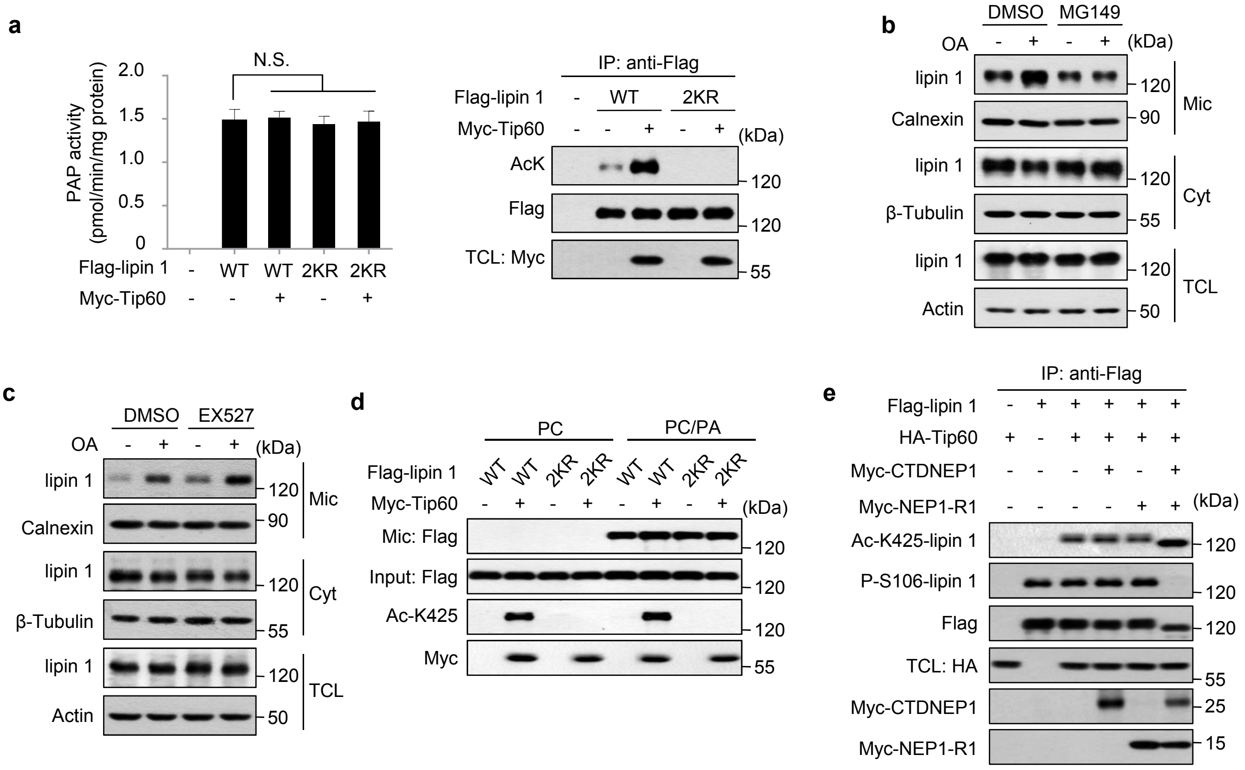


**Supplementary Fig. 4** Regulation of PAP activity and ER localization of lipin 1 by Tip60 or Sirt1 inhibitors. (**a**) Tip60-mediated acetylation of lipin 1 doesn't affect its PAP activity. Flag tagged WT or 2KR-lipin 1 were transfected with or without Tip60 in HEK293 cells, immunopurified with Flag antibody and measured for the $Mg^{2+}$-dependent PAP activity (left) ($n$ = 4 experiments). Representative western blots detecting the overexpressed proteins are shown (right). (**b**) Tip60 inhibitor MG149 blocks the ER localization of lipin 1. 3T3-L1 adipocytes were pretreated with DMSO or MG149 for 3 h and co-treated with BSA or OA for 3 h and analyzed by fractionation, followed by western blotting. (**c**) Sirt1 inhibitor EX527 promotes lipin 1 ER localization. 3T3-L1 adipocytes (differentiation day 8) were pretreated with DMSO or EX527 for 3 h and co-treated with or without OA for 3 h, analyzed by fractionation, followed by western blotting. (**d**) Binding of lipin 1 with artificial liposomes. Immuno-purified Flag-tagged WT-lipin 1 or 2KR mutant co-expressed with or without Tip60 in HEK293T cells were incubated with liposomes consist of phosphatidylcholine (PC) or PC with 20 mol% of phosphatidic acid (PA) (PC/PA) for 20 min. The lipin 1 protein binds with liposomes (mic) were purified by ultracentrifuge and analysed by western blotting. (**e**) Co-expression of CTDNEP1 and NEP1-R1 with lipin 1 leads to lipin 1 dephosphorylation and promotes its acetylation by Tip60. HEK293T cells were transfected with lipin 1, Tip60, CTDNEP1 and/or NEP1-R1, immunoprecipitated with antibody to Flag and immunoblotted as indicated. Error bars denote SEM. Statistical analysis was performed by ANOVA followed by Tukey in (**a**) (N.S., not significant). Uncropped blots can be found in Supplementary Fig. 6.

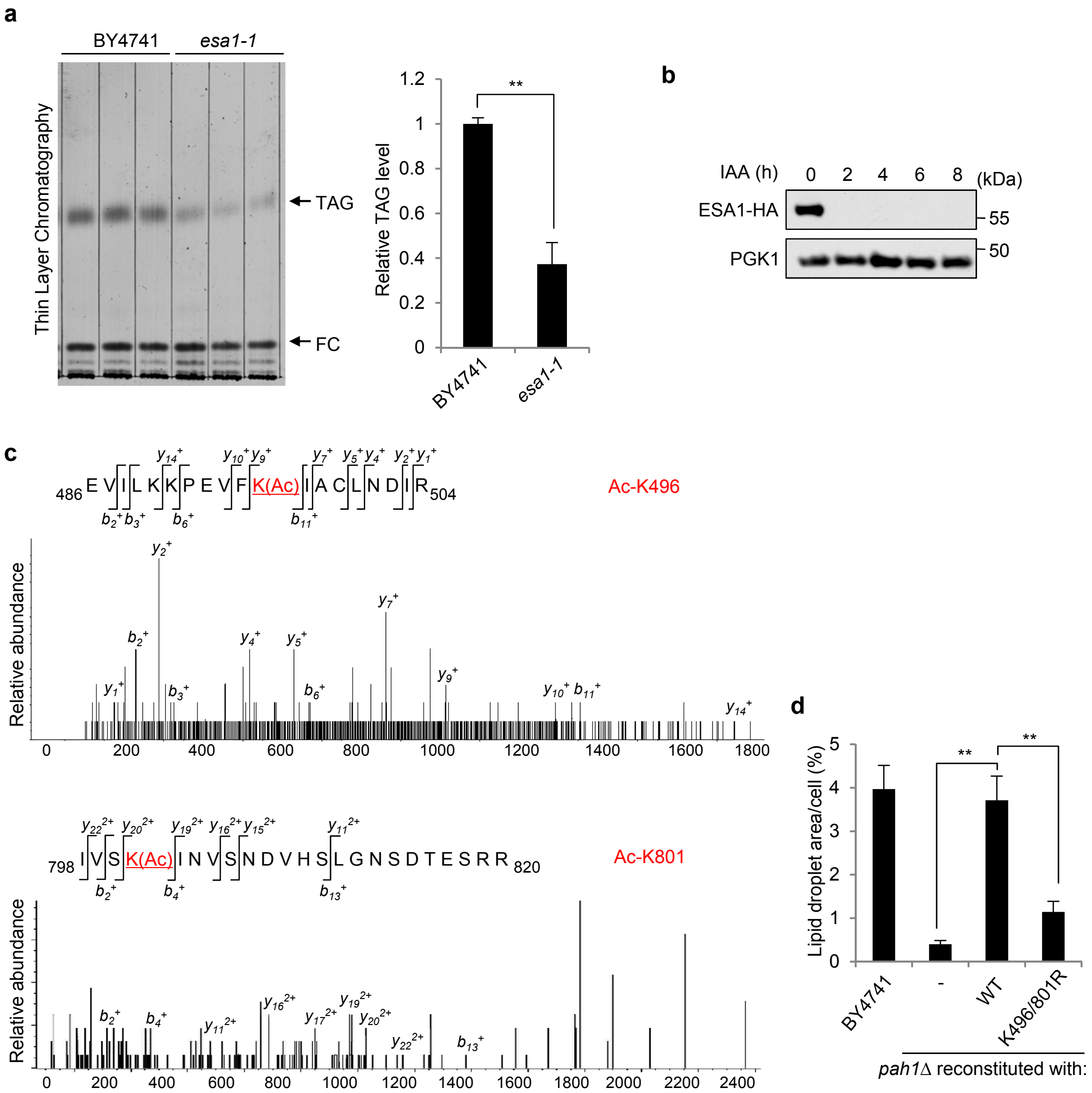


**Supplementary Fig. 5 Role of ESA1 (Yeast Tip60) in TAG accumulation in *S. cerevisiae* and PAH1 (Yeast Lipin 1) acetylation by ESA1.** (**a**) Impaired accumulation of TAG in *ESA1* loss of function mutant (*esa1-1*, an auxin-sensitive mutant strain of ESA1). TAG were extracted from cells grown in synthetic complete (SC) medium, separated by thin TLC, stained by iodine vapor and quantified. FC, free cholesterol (*n* = 3 experiments). (**b**) Rapid depletion of ESA1 by auxin indole-3-acetic acid (IAA) treatment in the auxin-sensitive mutant strain of ESA1. Cells grown in SC medium were collected for analysis at indicated time points after addition of 500 μM IAA. (**c**) Identification of K496 and K801 acetylation on PAH1 by liquid chromatography-mass spectrometry (LC-MS/MS) analysis. The MS/MS spectrum of the acetylated peptide EVILKKPEVFK(Ac)IACLNDIR (top) and IVSK(Ac)INVSNDVHSLGNSDTESRR (bottom) are shown. The labeled peaks show the masses of *y* or *b* ions of the acetylated peptides. (**d**) Percentage of lipid droplet area in WT (BY4741) or PAH1 knockout (*pah1Δ*) cells reconstituted with vector, WT-PAH1 or K496/801R-PAH1 (n = 100 cells quantified for each group). Error bars denote SEM. Statistical analysis was performed by two-tailed unpaired Student's *t*-test in (**a**) or by ANOVA followed by Tukey in (**d**), (**$P < 0.01$). Uncropped blots can be found in Supplementary Fig. 6.

**Supplementary Figure 6** Uncropped blots with size marker indications.

Figure 2e

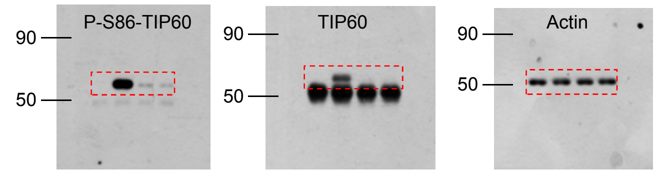


Figure 3b

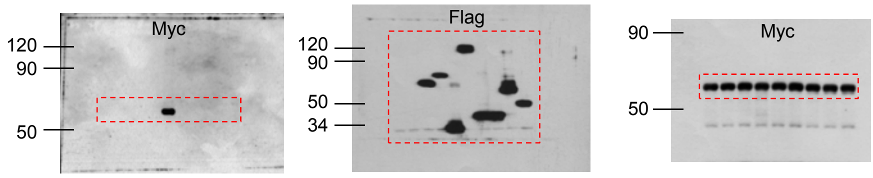


Figure 3c

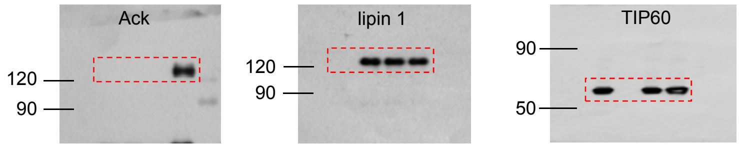


Figure 3d

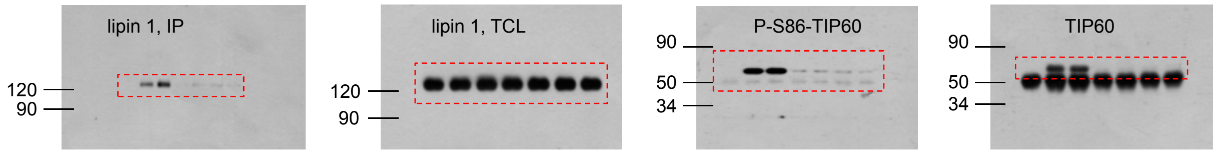


Figure 3e

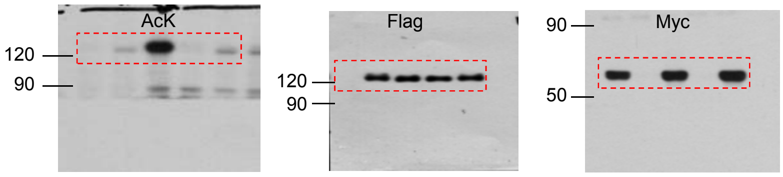


Figure 3f

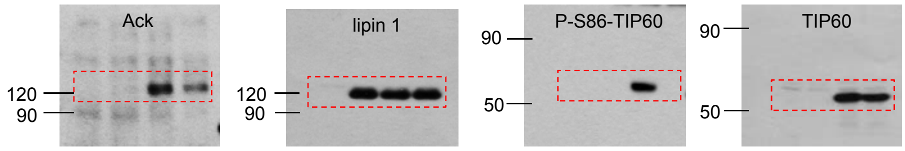


Figure 3g

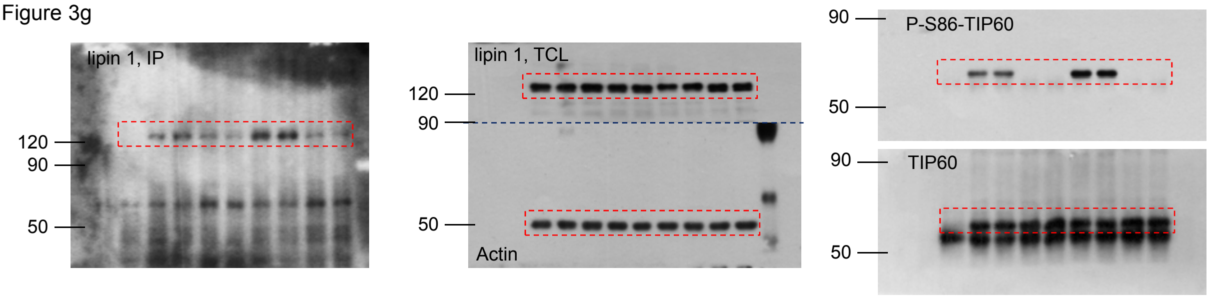

**Supplementary Figure 6** Uncropped blots with size marker indications (continued).

Figure 3k

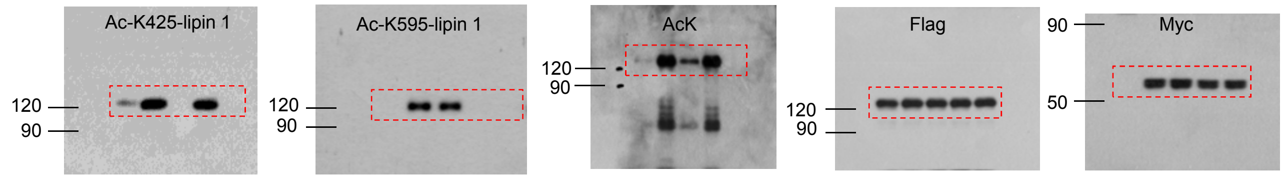


Figure 3l

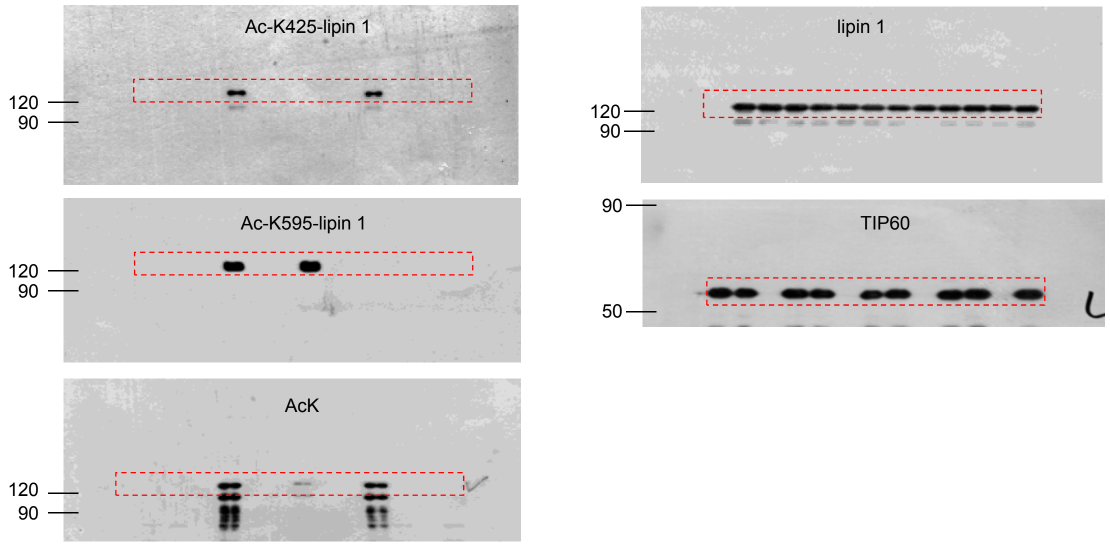


Figure 4a

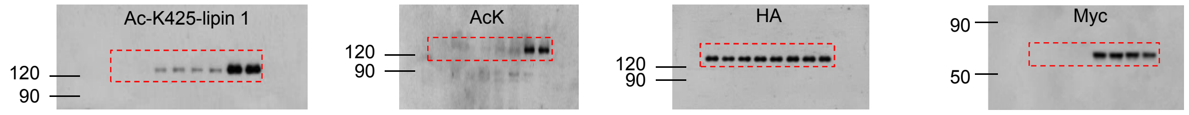


Figure 4b

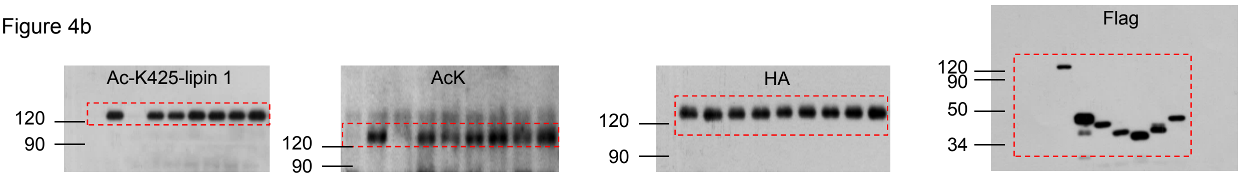


Figure 4c

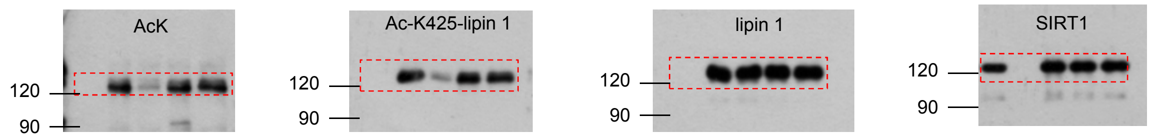


Figure 4d

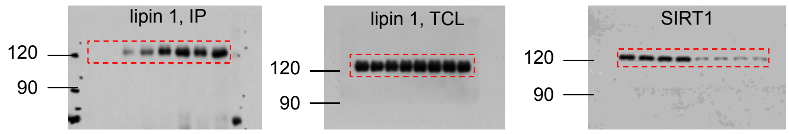


Figure 4e

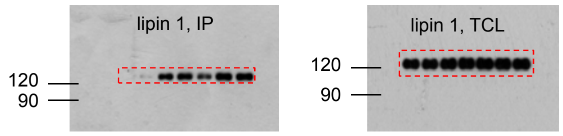

**Supplementary Figure 6** Uncropped blots with size marker indications (continued).

Figure 5b

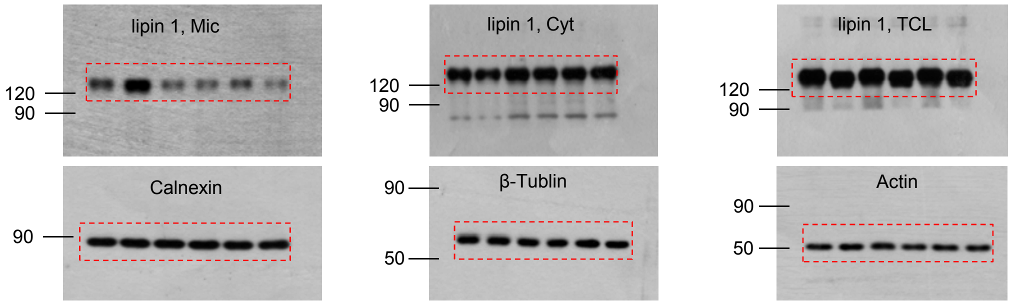


Figure 5c

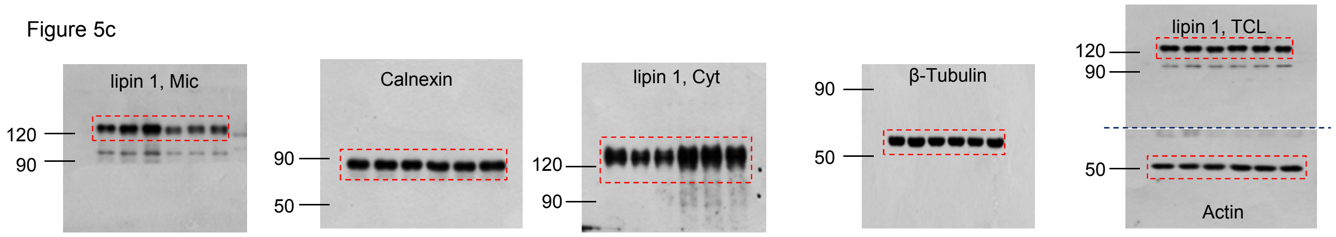


Figure 5d

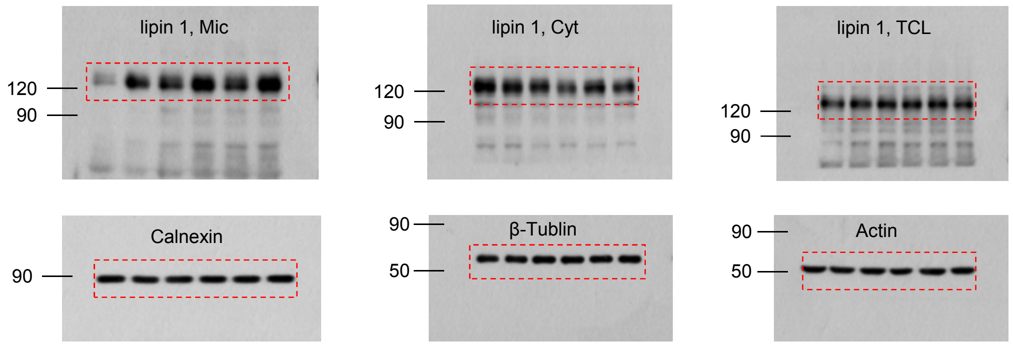


Figure 5e

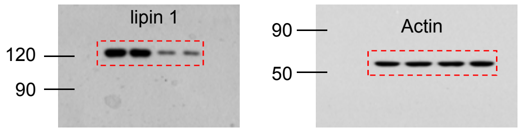


Figure 5f

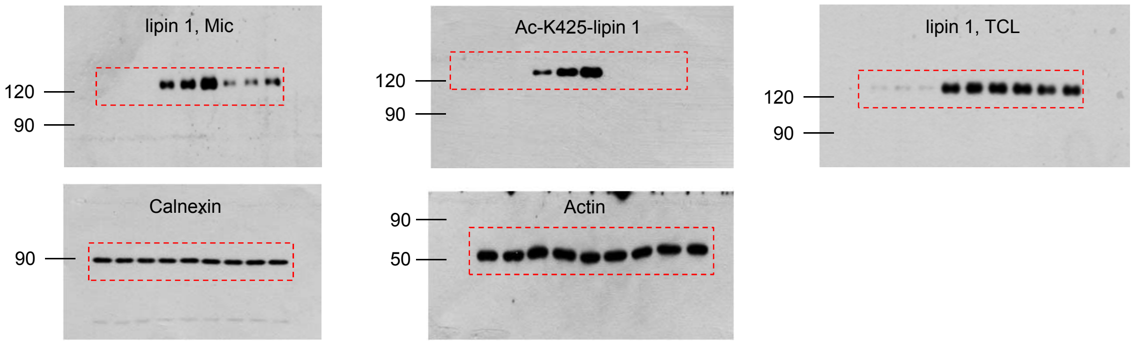

**Supplementary Figure 6** Uncropped blots with size marker indications (continued).

Figure 6a

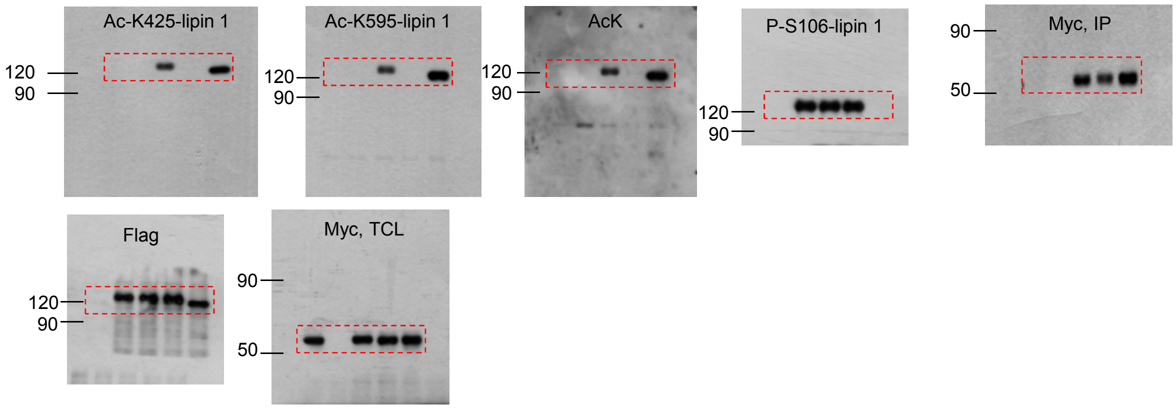


Figure 6b

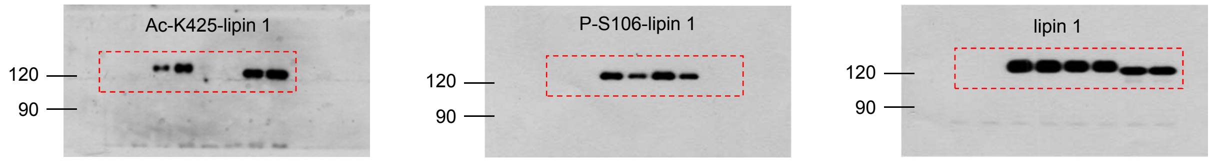


Figure 6c

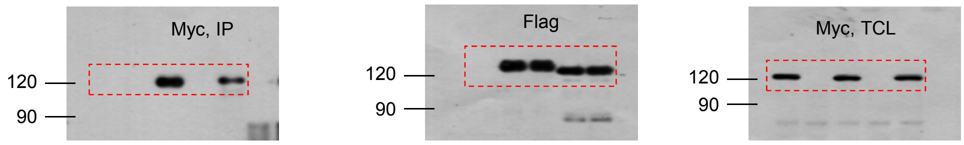


Figure 6d

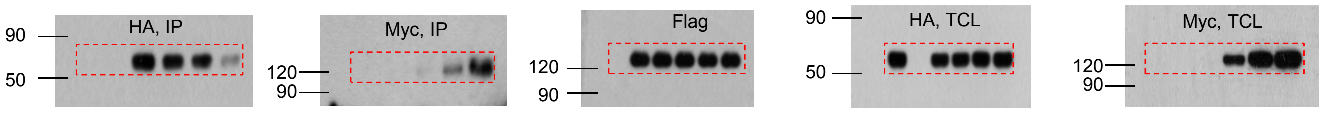


Figure 6e

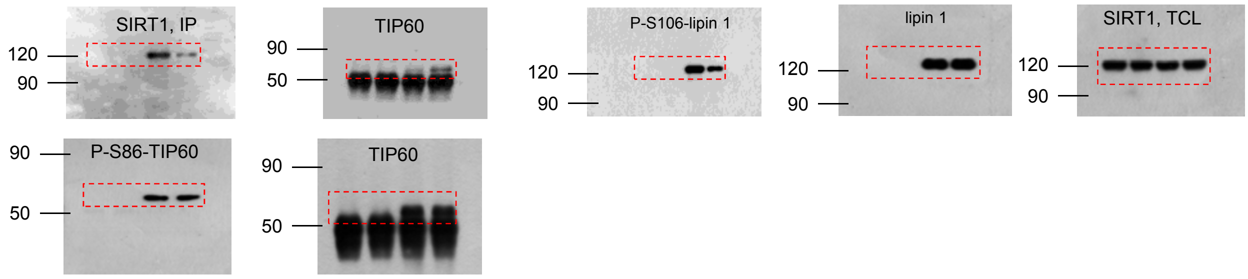


Figure 7e

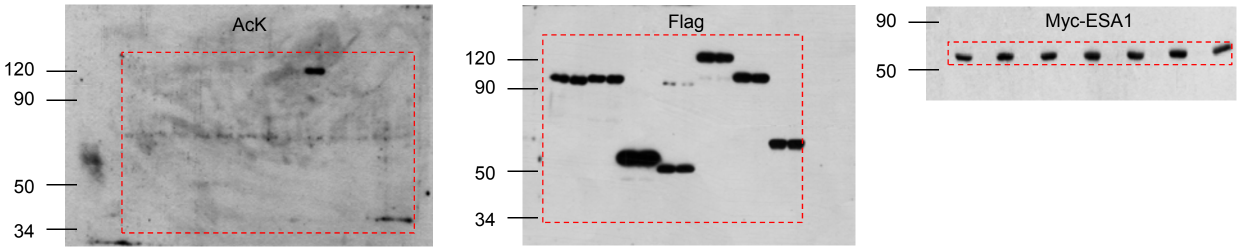

**Supplementary Figure 6** Uncropped blots with size marker indications (continued).

Figure 7f

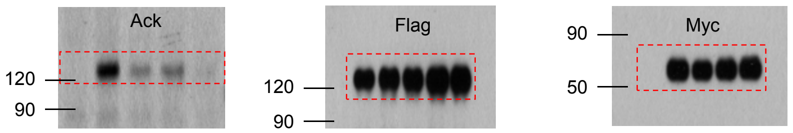


Figure 7g

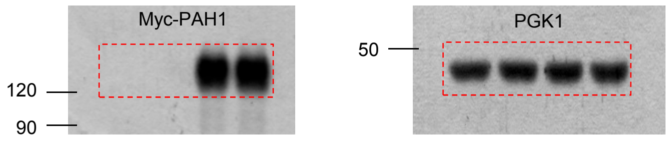


Supplementary Figure 1c

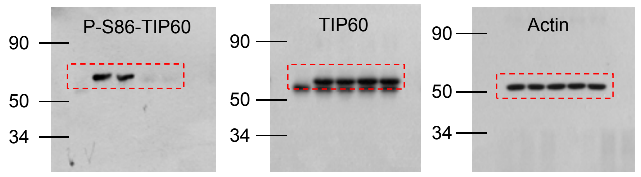


Supplementary Figure 1n

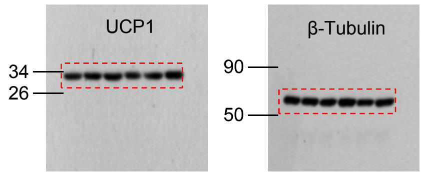


Supplementary Figure 2b

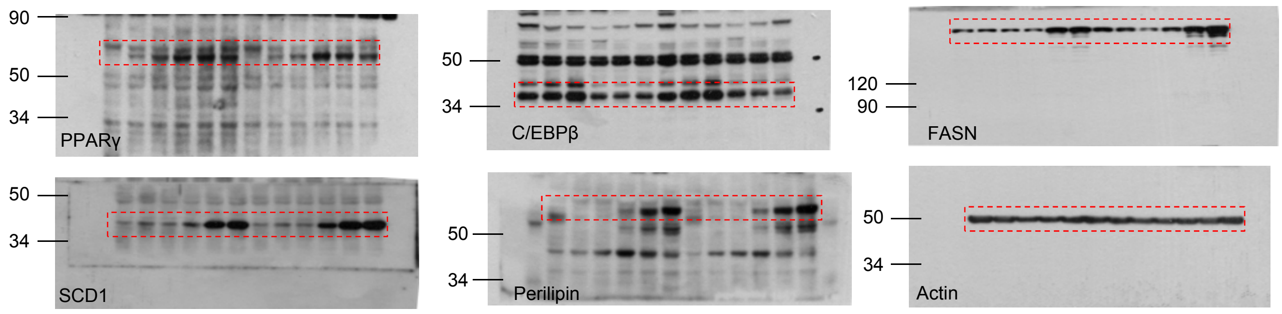


Supplementary Figure 2c

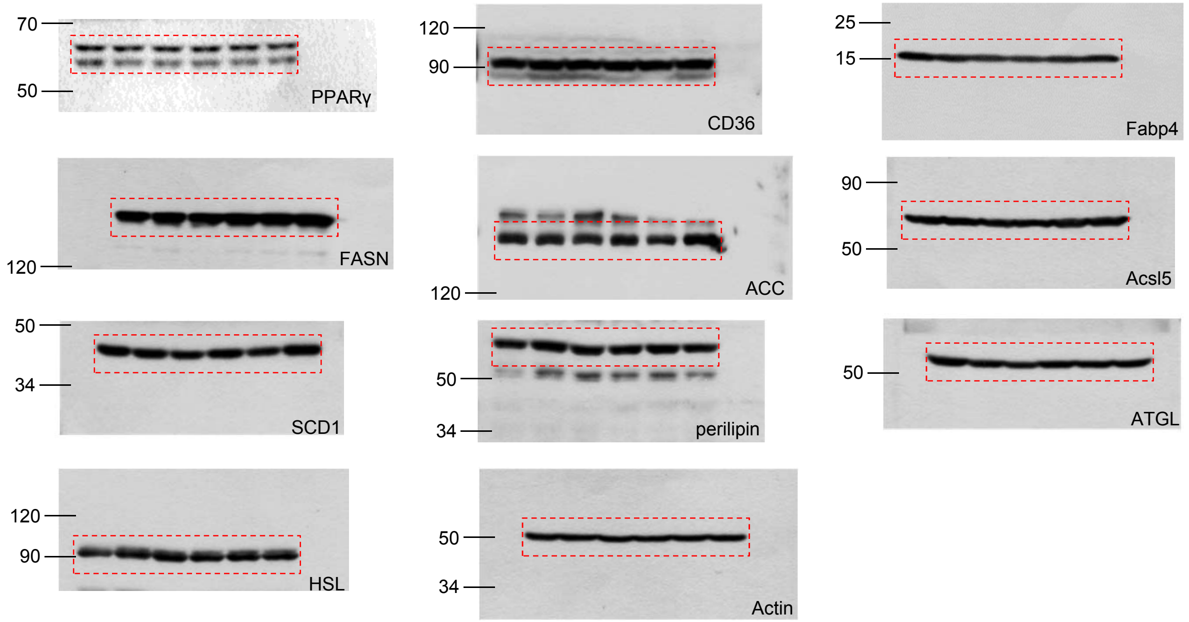

**Supplementary Figure 6** Uncropped blots with size marker indications (continued).

Supplementary Figure 3a

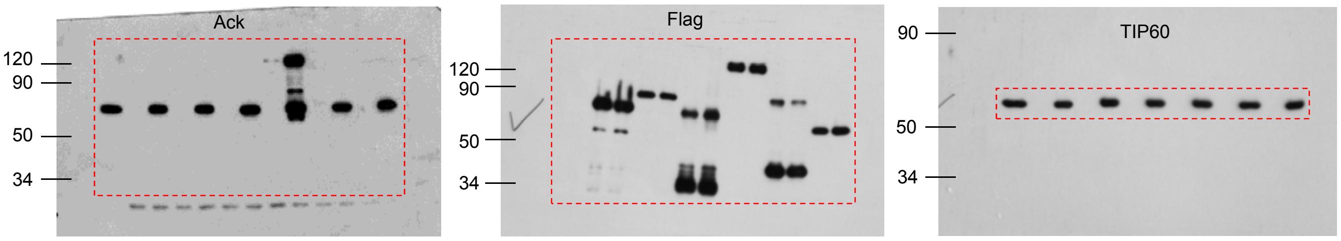


Supplementary Figure 3b

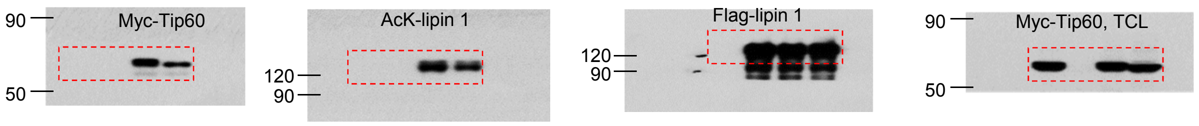


Supplementary Figure 4a

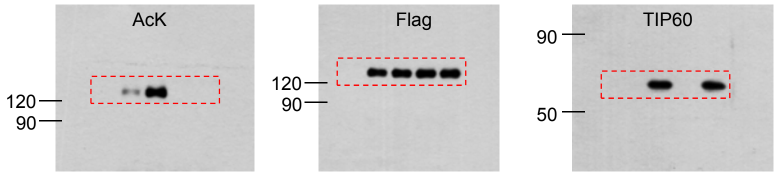


Supplementary Figure 4b

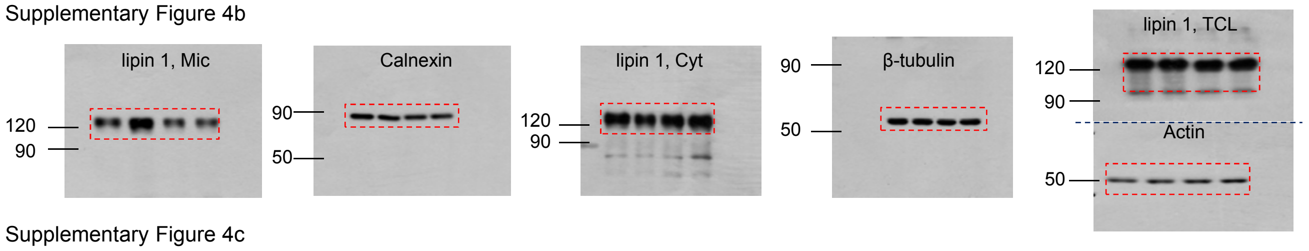


Supplementary Figure 4c

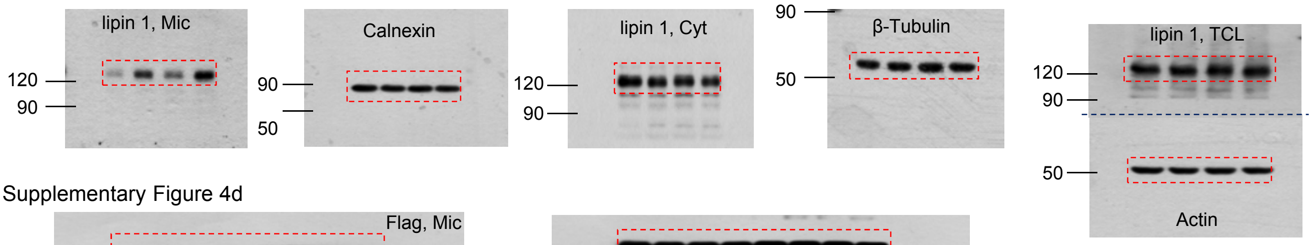


Supplementary Figure 4d

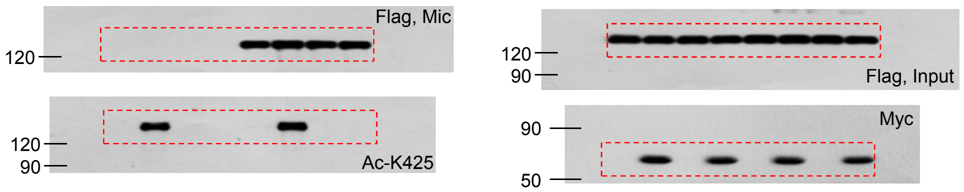


Supplementary Figure 4e

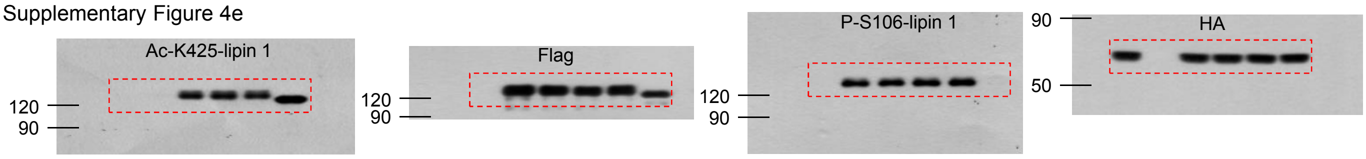


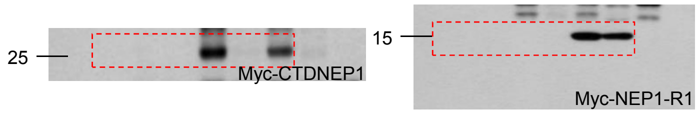


Supplementary Figure 5b

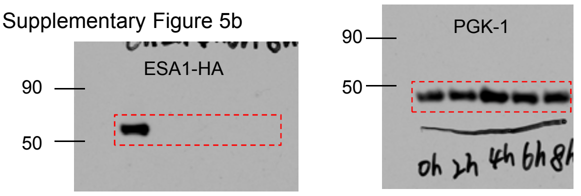

**Supplementary Table S1** Lipidomic Profiling of WT and *Tip60*$^{SA/SA}$ Mice eWAT by LC-MS.

| | WT-1 | WT-2 | WT-3 | S86A-1 | S86A-2 | S86A-3 | WT-mean | S86A-mean | fold change (S86A/WT) | *P*-values |
|---|---|---|---|---|---|---|---|---|---|---|
| DAG34:3(18:2/16:1) | 2.69600157 | 2.98579008 | 2.94658427 | 1.86371023 | 2.46053859 | 2.43990354 | 2.87612531 | 2.25471745 | 0.7839427 | 0.0495 |
| DAG36:4(18:2/18:2) | 10.1260838 | 10.1764994 | 11.6386407 | 8.1664648 | 9.59002327 | 8.30241527 | 10.6470746 | 8.68630111 | 0.81583922 | 0.0495 |
| DAG36:3(18:2/18:1) | 12.9123903 | 12.0256068 | 14.44311 | 10.3365188 | 12.38991 | 10.0793701 | 13.1270357 | 10.9352663 | 0.83303394 | 0.1266 |
| DAG34:3(16:2/18:1) | 0.14683384 | 0.12716175 | 0.14717302 | 0.12758368 | 0.1209062 | 0.11305434 | 0.14038954 | 0.12051474 | 0.85843105 | 0.1266 |
| DAG32:2(16:2/16:0) | 0.05039456 | 0.05955278 | 0.05785828 | 0.05134467 | 0.05503613 | 0.04083276 | 0.0559352 | 0.04907119 | 0.87728626 | 0.2752 |
| DAG36:2(18:1/18:1) | 10.2371479 | 9.34761668 | 11.3764287 | 8.94279858 | 8.96579359 | 8.40643043 | 10.3203978 | 8.7716742 | 0.84993567 | 0.1266 |
| DAG34:2(16:1/18:1) | 2.25326634 | 2.2382233 | 2.533602 | 1.70432722 | 2.17728282 | 2.33573726 | 2.34169721 | 2.0724491 | 0.8850201 | 0.2752 |
| GM3 18:0/18:0 | 0.00033346 | 0.00032316 | 0.00011842 | 0.00029621 | 0.00014103 | 0.00027589 | 0.00025835 | 0.00023771 | 0.92012072 | 0.5127 |
| PE38:4p | 0.06112815 | 0.04810464 | 0.06646267 | 0.05571995 | 0.05401127 | 0.05378806 | 0.05856515 | 0.05450642 | 0.93069722 | 0.5127 |
| DAG32:2(16:1/16:1) | 0.53459385 | 0.60819964 | 0.51648485 | 0.45904273 | 0.41021118 | 0.69588798 | 0.55309278 | 0.52171396 | 0.94326664 | 0.5127 |
| DAG38:4(18:1/20:3) | 0.17188626 | 0.16963364 | 0.17735656 | 0.19837691 | 0.13232093 | 0.16241942 | 0.17295882 | 0.16437242 | 0.95035581 | 0.5127 |
| DAG | 65.9151289 | 64.8768009 | 66.9346427 | 60.6302128 | 63.5302239 | 63.5996522 | 65.9088575 | 62.5866963 | 0.94959462 | 0.0495 |
| DAG32:1(14:0/18:1) | 0.34410147 | 0.37154682 | 0.35059432 | 0.33027347 | 0.32441336 | 0.3635994 | 0.35541421 | 0.33942874 | 0.955023 | 0.2752 |
| PI 34:1 | 0.02141972 | 0.01464336 | 0.02241084 | 0.02305576 | 0.01516732 | 0.018123 | 0.01949131 | 0.01878202 | 0.96361016 | 0.8273 |
| PS 34:1 | 0.0103786 | 0.00902592 | 0.00868296 | 0.00931238 | 0.00885628 | 0.00892045 | 0.00936249 | 0.0090297 | 0.9644552 | 0.8273 |
| PE34:1p | 0.0232403 | 0.02413007 | 0.02163804 | 0.02709045 | 0.01756018 | 0.02307818 | 0.0230028 | 0.02257627 | 0.98145749 | 0.8273 |
| DAG32:2(18:2/14:0) | 0.64613084 | 0.78782064 | 0.69426741 | 0.59449606 | 0.61734372 | 0.87777549 | 0.7094063 | 0.69653842 | 0.98186106 | 0.5127 |
| PE36:3p | 0.02339785 | 0.02474244 | 0.01852521 | 0.02135983 | 0.02080886 | 0.02345857 | 0.02222183 | 0.02187575 | 0.98442597 | 0.8273 |
| PC36:2p | 0.00397417 | 0.00529339 | 0.00534223 | 0.00539701 | 0.0051523 | 0.00387035 | 0.00486993 | 0.00480656 | 0.98698625 | 0.8273 |
| DAG30:1(14:0/16:1) | 0.08989291 | 0.11325375 | 0.08343669 | 0.07118227 | 0.07582105 | 0.13621815 | 0.09552778 | 0.09440716 | 0.9882691 | 0.5127 |
| PE36:4p | 0.09168151 | 0.0921776 | 0.0950782 | 0.09403041 | 0.08511005 | 0.09744999 | 0.0929791 | 0.09219682 | 0.99158642 | 0.8273 |
| PI 40:6 | 0.00650364 | 0.00507054 | 0.01269713 | 0.00988526 | 0.00745521 | 0.00682107 | 0.00809043 | 0.00805385 | 0.99547758 | 0.5127 |
| PC32:2 | 0.01042903 | 0.00889433 | 0.02011728 | 0.01357604 | 0.01138376 | 0.01495779 | 0.01314688 | 0.01330586 | 1.01209287 | 0.5127 |
| DAG40:6(18:1/22:5) | 0.18904977 | 0.1608413 | 0.12494203 | 0.18126212 | 0.1381758 | 0.16333117 | 0.1582777 | 0.16092303 | 1.01671322 | 0.8273 |
| GM3 18:1/18:1 | 0.00013349 | 0.00023514 | 6.7256E-05 | 4.9519E-05 | 0.00024567 | 0.00015351 | 0.0001453 | 0.00014956 | 1.02936812 | 0.8273 |
| LPA18:1 | 0.04904401 | 0.04654232 | 0.06346141 | 0.06986236 | 0.04946468 | 0.04514659 | 0.05301592 | 0.05482454 | 1.03411479 | 0.8273 |
| PE36:4 | 0.10999395 | 0.07825909 | 0.06606623 | 0.10371774 | 0.07042086 | 0.08905456 | 0.08477309 | 0.08773105 | 1.03489271 | 0.8273 |
| GM3 18:1/22:0 | 0.00106696 | 0.00196658 | 0.00062403 | 0.0011338 | 0.00098266 | 0.00171167 | 0.00121919 | 0.00127604 | 1.04663257 | 0.8273 |
| DAG34:1(16:1/18:0) | 0.22991668 | 0.20087242 | 0.1937095 | 0.18904151 | 0.19701993 | 0.27029593 | 0.2081662 | 0.21878579 | 1.05101495 | 0.8273 |
| PC40:5 | 0.01189307 | 0.01274124 | 0.02202173 | 0.02333288 | 0.01058309 | 0.0152403 | 0.01555201 | 0.01638543 | 1.05358867 | 0.8273 |
| PI 36:1 | 0.01109958 | 0.00867773 | 0.01542865 | 0.01712751 | 0.00967335 | 0.01070001 | 0.01173532 | 0.01250029 | 1.06518563 | 0.8273 |
| GM3 18:0/22:0 | 0.00036752 | 0.00055741 | 0.0002699 | 0.00068918 | 0.00035091 | 0.00024477 | 0.00039828 | 0.00042829 | 1.07535122 | 0.8273 |
| DAG34:1(16:0/18:1) | 6.23308958 | 5.92479521 | 5.35644496 | 6.36121595 | 6.00625367 | 6.52835428 | 5.83810992 | 6.29860797 | 1.07887793 | 0.1266 |
| PE40:4p | 0.07799852 | 0.08290325 | 0.06282909 | 0.08280374 | 0.08473791 | 0.07552452 | 0.07457695 | 0.08102206 | 1.0864222 | 0.5127 |
| DAG32:1(16:1/16:0) | 0.86588153 | 1.08622292 | 0.83282202 | 0.92215878 | 0.87338113 | 1.2474694 | 0.92830882 | 1.01433644 | 1.09267133 | 0.2752 |
| PE38:6p | 0.14118468 | 0.15867086 | 0.11567863 | 0.17990586 | 0.13395779 | 0.14645243 | 0.13851139 | 0.15343869 | 1.1077695 | 0.5127 |
| PS 40:7 | 0.03865234 | 0.03445199 | 0.02999675 | 0.05389696 | 0.03300327 | 0.02755974 | 0.03436703 | 0.03815333 | 1.11017242 | 0.8273 |
| CE:18:1 | 0.27916352 | 0.38990964 | 0.30718565 | 0.39488828 | 0.32498712 | 0.3640371 | 0.3254196 | 0.36130417 | 1.11027167 | 0.2752 |
| PI 34:2 | 0.02170286 | 0.01394911 | 0.01492335 | 0.02207264 | 0.01677198 | 0.01760262 | 0.01685844 | 0.01881574 | 1.11610228 | 0.2752 |
| DAG34:2(16:0/18:2) | 12.1858046 | 12.1649974 | 10.7147984 | 12.9008248 | 12.9505898 | 13.6791171 | 11.6885335 | 13.1768439 | 1.12733081 | 0.0495 |
| DAG36:1(18:1/18:0) | 0.92971966 | 1.00450286 | 0.78551744 | 0.99171675 | 0.92327902 | 1.17234921 | 0.90657999 | 1.02911499 | 1.13516182 | 0.5127 |
| DAG36:2(18:2/18:0) | 1.7348532 | 1.86183806 | 1.52727893 | 1.95741566 | 1.7289516 | 2.15409606 | 1.70799006 | 1.94682111 | 1.13983164 | 0.2752 |
| PA34:1 | 0.02051844 | 0.018021 | 0.01596654 | 0.02631283 | 0.01788765 | 0.0179992 | 0.01816866 | 0.02073323 | 1.14115338 | 0.8273 |
| PC34:1 | 0.29730082 | 0.33002332 | 0.32446567 | 0.40637769 | 0.31253223 | 0.36801758 | 0.31726327 | 0.36230917 | 1.14198272 | 0.2752 |
| PE38:5p | 0.06016842 | 0.06171003 | 0.06037305 | 0.07640937 | 0.06716071 | 0.06559881 | 0.0607505 | 0.06972296 | 1.14769362 | 0.0495 |
| LPE18:1 | 0.03597982 | 0.02739321 | 0.03838407 | 0.0480991 | 0.0308177 | 0.03788274 | 0.03391903 | 0.03893318 | 1.14782705 | 0.5127 |
| PE42:1p | 0.0109874 | 0.0108332 | 0.00959291 | 0.01322153 | 0.00820899 | 0.01488301 | 0.01047117 | 0.01210451 | 1.15598462 | 0.5127 |
| PC36:4 | 0.15754733 | 0.1514319 | 0.13071968 | 0.20381965 | 0.13664875 | 0.16919402 | 0.1465663 | 0.16988747 | 1.15911687 | 0.2752 |
| PC32:1 | 0.08280728 | 0.09855956 | 0.11638809 | 0.11932923 | 0.1088406 | 0.12039846 | 0.09925164 | 0.11618943 | 1.17065502 | 0.1266 |
| PC38:4 | 0.09778313 | 0.12217969 | 0.11560635 | 0.16452925 | 0.1091851 | 0.11995048 | 0.11185639 | 0.13122161 | 1.17312573 | 0.5127 |
| LPE16:0 | 0.01733326 | 0.01874128 | 0.00875532 | 0.01585485 | 0.01566655 | 0.02172351 | 0.01494329 | 0.0177483 | 1.18771074 | 0.8273 |
| PC38:5 | 0.04803209 | 0.04860675 | 0.0662931 | 0.08451011 | 0.05146331 | 0.05754461 | 0.05431065 | 0.06450601 | 1.18772315 | 0.2752 |
| PC38:3 | 0.06046441 | 0.07295956 | 0.05829457 | 0.09680016 | 0.06305905 | 0.06996376 | 0.06390618 | 0.07660766 | 1.19875197 | 0.2752 |
| CE:18:0 | 0.11753994 | 0.10072028 | 0.05217228 | 0.10740095 | 0.08978682 | 0.12950327 | 0.09014417 | 0.10889701 | 1.20803173 | 0.5127 |
| DAG34:0(16:0/18:0) | 0.64157852 | 0.63076337 | 0.48325407 | 0.73017336 | 0.5670922 | 0.83048755 | 0.58519865 | 0.70925103 | 1.21198337 | 0.2752 |
| DAG38:4(18:0/20:4) | 0.25691075 | 0.25118961 | 0.19121234 | 0.32366406 | 0.25648469 | 0.26788981 | 0.23310423 | 0.28267952 | 1.21267434 | 0.1266 |
| GM3 18:1/18:0 | 0.00060045 | 0.00079333 | 0.00040473 | 0.00093663 | 0.00045604 | 0.00079472 | 0.0005995 | 0.00072913 | 1.21621913 | 0.2752 |
| PE34:1 | 0.07910394 | 0.07701804 | 0.0493598 | 0.08962424 | 0.07117649 | 0.08924905 | 0.06849393 | 0.08334992 | 1.21689512 | 0.2752 |
| PC40:6 | 0.03173789 | 0.04146118 | 0.04230668 | 0.06241112 | 0.03610499 | 0.04306326 | 0.03850191 | 0.04719312 | 1.22573451 | 0.2752 |
| LPI16:1 | 0.00267747 | 0.00237151 | 0.00133006 | 0.00244902 | 0.00240483 | 0.00300775 | 0.00212634 | 0.00262053 | 1.23241205 | 0.2752 |
| PE42:2p | 0.01018764 | 0.01407858 | 0.00946278 | 0.01211499 | 0.01392789 | 0.01563815 | 0.011243 | 0.01389368 | 1.23576248 | 0.2752 |
| GM3 | 0.00777145 | 0.00960109 | 0.0050603 | 0.01093819 | 0.00768582 | 0.00920113 | 0.00747761 | 0.00927505 | 1.24037563 | 0.5127 |
| PC36:3 | 0.23305945 | 0.26746142 | 0.19901587 | 0.36179311 | 0.24210051 | 0.26425085 | 0.23317891 | 0.28938149 | 1.24102769 | 0.2752 |
| PC38:6 | 0.04570061 | 0.04352616 | 0.0454238 | 0.06889029 | 0.04718164 | 0.05145461 | 0.04488353 | 0.05584218 | 1.24415754 | 0.0495 |
| PC34:3 | 0.02480465 | 0.02997852 | 0.02743743 | 0.04152165 | 0.02593833 | 0.03552366 | 0.02740687 | 0.03432788 | 1.25252846 | 0.2752 |
| PC36:1 | 0.19936475 | 0.27758406 | 0.28597147 | 0.37572139 | 0.26632881 | 0.31601599 | 0.25430676 | 0.3193554 | 1.25578808 | 0.2752 |
| PC32:2e | 0.00681293 | 0.00723226 | 0.00710754 | 0.00946563 | 0.00807659 | 0.00907129 | 0.00705091 | 0.00887117 | 1.25815923 | 0.0495 |
| PE34:2p | 0.0178301 | 0.02041843 | 0.0145069 | 0.02664448 | 0.02385715 | 0.01601854 | 0.01758514 | 0.02217339 | 1.26091595 | 0.2752 |
| GM3 18:1/16:0 | 0.00323331 | 0.00355201 | 0.00227666 | 0.00458123 | 0.00326139 | 0.00360587 | 0.00302066 | 0.00381616 | 1.26335283 | 0.1266 |
| CE | 0.87459623 | 1.04764967 | 0.90688781 | 1.304008 | 1.01151165 | 1.26852945 | 0.94304457 | 1.19468304 | 1.26683624 | 0.1266 |
| CE:18:2 | 0.23817311 | 0.29529966 | 0.31304777 | 0.40791522 | 0.32658264 | 0.33854421 | 0.28217351 | 0.35768069 | 1.26759132 | 0.0495 |
| PC32:0 | 0.15868396 | 0.19515276 | 0.16287755 | 0.24368702 | 0.19529817 | 0.2193724 | 0.17223809 | 0.21945253 | 1.27412313 | 0.0495 |
| LPS16:0 | 0.00167664 | 0.00158812 | 0.00118861 | 0.00186235 | 0.00148596 | 0.00233148 | 0.00148446 | 0.00189326 | 1.27539006 | 0.2752 |

| | | | | | | | | | | |
|---|---|---|---|---|---|---|---|---|---|---|
| **PS 36:1** | 0.26575004 | 0.28069763 | 0.26121344 | 0.37566097 | 0.3248546 | 0.33347693 | 0.26922037 | 0.34466417 | 1.28023064 | 0.0495 |
| **PA38:4** | 0.01742665 | 0.0301587 | 0.01907011 | 0.03198939 | 0.02766613 | 0.02581511 | 0.02221849 | 0.02849021 | 1.28227495 | 0.2752 |
| **PE36:1p** | 0.02133501 | 0.01763015 | 0.01632569 | 0.02686254 | 0.02099492 | 0.02307818 | 0.01843028 | 0.02364521 | 1.28295469 | 0.1266 |
| **PA36:1** | 0.01018401 | 0.01462002 | 0.01431876 | 0.01870623 | 0.01583423 | 0.01579574 | 0.01304093 | 0.01677873 | 1.28662075 | 0.0495 |
| **PC34:0p** | 0.01479149 | 0.01880462 | 0.01721205 | 0.02146402 | 0.02572771 | 0.01818271 | 0.01693605 | 0.02179148 | 1.28669187 | 0.1266 |
| **PS 38:4** | 0.16050706 | 0.15008294 | 0.12305863 | 0.23007736 | 0.17472524 | 0.15363543 | 0.14454954 | 0.18614601 | 1.28776617 | 0.1266 |
| **PA32:0** | 0.00294368 | 0.00309704 | 0.00228447 | 0.00350725 | 0.00307877 | 0.00415413 | 0.00277506 | 0.00358005 | 1.29007773 | 0.1266 |
| **PS 40:4** | 0.03134114 | 0.03187226 | 0.02831646 | 0.04254354 | 0.0371876 | 0.03850973 | 0.03050996 | 0.03941362 | 1.29182817 | 0.0495 |
| **PC36:1p** | 0.02525194 | 0.03456481 | 0.03073434 | 0.04276585 | 0.0362443 | 0.03802245 | 0.0301837 | 0.03901086 | 1.29244824 | 0.0495 |
| **PE** | 2.26432032 | 2.15208506 | 1.62000986 | 3.02964008 | 2.26552494 | 2.50953958 | 2.01213842 | 2.6015682 | 1.292937 | 0.0495 |
| **PE38:5** | 0.09979917 | 0.07609469 | 0.06179738 | 0.12860178 | 0.09274664 | 0.08600808 | 0.07923041 | 0.10245217 | 1.29309142 | 0.2752 |
| **PC36:3p** | 0.0057074 | 0.00517029 | 0.00531904 | 0.00560463 | 0.01023489 | 0.00512024 | 0.00539891 | 0.00698659 | 1.29407329 | 0.8273 |
| **PS 38:5** | 0.02078148 | 0.02014173 | 0.01828177 | 0.03354827 | 0.02348908 | 0.01968553 | 0.01973499 | 0.02557429 | 1.29588571 | 0.2752 |
| **PC36:0p** | 0.01341699 | 0.01686597 | 0.01347232 | 0.01847453 | 0.0195999 | 0.01870717 | 0.01458509 | 0.0189272 | 1.29770838 | 0.0495 |
| **LPA** | 0.31674626 | 0.33266593 | 0.30328472 | 0.52821212 | 0.37551219 | 0.33657932 | 0.31756564 | 0.41343454 | 1.30188691 | 0.0495 |
| **LPS18:1** | 0.01142842 | 0.01208017 | 0.01154875 | 0.01524592 | 0.01495176 | 0.01550042 | 0.01168578 | 0.0152327 | 1.30352432 | 0.0495 |
| **PS 38:3** | 0.07720206 | 0.07501516 | 0.0652268 | 0.11985092 | 0.08662007 | 0.07877226 | 0.07248134 | 0.09508108 | 1.31180082 | 0.0495 |
| **PI 36:4** | 0.10674988 | 0.12364191 | 0.08766514 | 0.18531211 | 0.09855191 | 0.13365965 | 0.10601898 | 0.13917456 | 1.31273254 | 0.2752 |
| **PE40:4** | 0.02610773 | 0.03866094 | 0.02591278 | 0.03942351 | 0.03511172 | 0.04463171 | 0.03022715 | 0.03972231 | 1.31412697 | 0.1266 |
| **PA38:5** | 0.00437367 | 0.00572669 | 0.00348464 | 0.00579968 | 0.00549828 | 0.00659827 | 0.00452833 | 0.00596541 | 1.31735203 | 0.1266 |
| **GM3 18:1/20:0** | 0.00076745 | 0.00055786 | 0.00048896 | 0.00078822 | 0.00063224 | 0.00097794 | 0.00060476 | 0.00079947 | 1.32196228 | 0.1266 |
| **PE34:2** | 0.21632291 | 0.19022409 | 0.12772957 | 0.26093265 | 0.19826195 | 0.24829596 | 0.17809219 | 0.23583019 | 1.32420285 | 0.1266 |
| **PE34:0** | 0.01273754 | 0.01082158 | 0.01010594 | 0.01696032 | 0.01069069 | 0.01697395 | 0.01122169 | 0.01487499 | 1.3255569 | 0.2752 |
| **PC** | 2.96161219 | 3.67384598 | 2.99950282 | 5.13360384 | 3.57529742 | 4.08410988 | 3.21165366 | 4.26433705 | 1.3277699 | 0.1266 |
| **DAG32:0(16:0/16:0)** | 2.14730821 | 2.31798293 | 1.50567851 | 2.82752029 | 2.21253409 | 2.88954185 | 1.99032321 | 2.64319874 | 1.32802488 | 0.1266 |
| **PS** | 1.46951122 | 1.51202813 | 1.22851638 | 2.25659442 | 1.74320487 | 1.5965504 | 1.40335191 | 1.8654499 | 1.32928162 | 0.0495 |
| **PA32:2** | 0.0030195 | 0.00433362 | 0.00272443 | 0.00459546 | 0.00382058 | 0.00505193 | 0.00335918 | 0.00448933 | 1.33643503 | 0.1266 |
| **PE38:6** | 0.12112934 | 0.11660916 | 0.06335176 | 0.17044962 | 0.11869324 | 0.11385469 | 0.10036342 | 0.13433252 | 1.33846095 | 0.5127 |
| **PC32:1e** | 0.01243067 | 0.01501847 | 0.0138902 | 0.02096549 | 0.01667536 | 0.01769923 | 0.01377978 | 0.0184467 | 1.33867831 | 0.0495 |
| **PS 40:6** | 0.53507993 | 0.5615751 | 0.41339473 | 0.87442963 | 0.5999648 | 0.55647013 | 0.50334992 | 0.67695485 | 1.3448991 | 0.1266 |
| **PG36:2** | 0.00326429 | 0.00295394 | 0.00284643 | 0.0037927 | 0.00342389 | 0.004998 | 0.00302155 | 0.00407153 | 1.34749525 | 0.0495 |
| **PI 36:3** | 0.04468254 | 0.04454816 | 0.02903636 | 0.07224171 | 0.04187065 | 0.04529827 | 0.03942235 | 0.05313688 | 1.34788702 | 0.2752 |
| **LPE** | 0.13699527 | 0.12468815 | 0.10337245 | 0.19073259 | 0.13744424 | 0.16451627 | 0.12168529 | 0.16423103 | 1.34963755 | 0.0495 |
| **PE32:0** | 0.00748249 | 0.00479496 | 0.00505392 | 0.00793022 | 0.00649168 | 0.00897045 | 0.00577712 | 0.00779745 | 1.34971157 | 0.1266 |
| **PE38:4** | 0.18751255 | 0.17537765 | 0.13122904 | 0.28559529 | 0.1862404 | 0.19528037 | 0.16470641 | 0.22237202 | 1.35011149 | 0.1266 |
| **PE36:3** | 0.18591282 | 0.15945034 | 0.1154202 | 0.25256963 | 0.17440764 | 0.19527178 | 0.15359445 | 0.20741635 | 1.35041559 | 0.1266 |
| **LPI20:4** | 0.00573443 | 0.00835278 | 0.00601113 | 0.01086641 | 0.00832719 | 0.00803223 | 0.00669944 | 0.00907528 | 1.35463102 | 0.2752 |
| **LPA16:0** | 0.05200857 | 0.0510788 | 0.05309402 | 0.08496336 | 0.06893552 | 0.05781741 | 0.05206046 | 0.07057209 | 1.35557946 | 0.0495 |
| **PC34:1p** | 0.00893477 | 0.00994045 | 0.00884951 | 0.01245466 | 0.01232372 | 0.01290136 | 0.00924158 | 0.01255992 | 1.35906633 | 0.0495 |
| **PE38:7** | 0.02786019 | 0.02119556 | 0.0136054 | 0.03347164 | 0.02252333 | 0.0293769 | 0.02088705 | 0.02845729 | 1.36243705 | 0.1266 |
| **GM3 18:1/20:1** | 0.00053442 | 0.00055864 | 0.0003205 | 0.00103491 | 0.00052711 | 0.00036659 | 0.00047119 | 0.00064287 | 1.36436549 | 0.8273 |
| **PC40:7** | 0.01018951 | 0.01705006 | 0.01646896 | 0.02275092 | 0.01580523 | 0.02116624 | 0.01456951 | 0.01990746 | 1.36637837 | 0.2752 |
| **PE40:5** | 0.04076001 | 0.04051905 | 0.02824634 | 0.05835235 | 0.04064727 | 0.05091902 | 0.03650847 | 0.04997288 | 1.36880243 | 0.1266 |
| **PS 34:2** | 0.01035234 | 0.00830408 | 0.00654447 | 0.0129644 | 0.01141584 | 0.01024231 | 0.0084003 | 0.01154085 | 1.37386182 | 0.1266 |
| **PG36:3** | 0.0013367 | 0.00234436 | 0.00104151 | 0.00222171 | 0.0018159 | 0.00245344 | 0.00157419 | 0.00216368 | 1.37447172 | 0.2752 |
| **PS 40:5** | 0.13230814 | 0.13308907 | 0.09947614 | 0.21377487 | 0.15109808 | 0.13957855 | 0.12162445 | 0.1681505 | 1.38253862 | 0.0495 |
| **LPE16:1** | 0.0091087 | 0.00864656 | 0.00707902 | 0.01132238 | 0.01163436 | 0.0115512 | 0.00827809 | 0.01150265 | 1.38952835 | 0.0495 |
| **PI 36:2** | 0.04446829 | 0.03386174 | 0.03126179 | 0.06751556 | 0.03885555 | 0.04599341 | 0.03653061 | 0.05078818 | 1.39029111 | 0.1266 |
| **PE32:2** | 0.01226004 | 0.01268438 | 0.00816476 | 0.01651783 | 0.0164123 | 0.01316387 | 0.01103639 | 0.01536467 | 1.3921819 | 0.0495 |
| **PE40:6p** | 0.05873822 | 0.05892622 | 0.04754301 | 0.08456417 | 0.06660534 | 0.07895999 | 0.05506915 | 0.07670983 | 1.39297294 | 0.0495 |
| **PA** | 0.1231879 | 0.17595874 | 0.12748246 | 0.22769695 | 0.18911846 | 0.18877378 | 0.1422097 | 0.20186306 | 1.41947461 | 0.0495 |
| **PC34:2** | 0.59032488 | 0.75423806 | 0.53848179 | 1.08717398 | 0.71611749 | 0.84385478 | 0.62768158 | 0.88238208 | 1.4057798 | 0.1266 |
| **PE40:6** | 0.1279793 | 0.12821491 | 0.08174684 | 0.20610769 | 0.12250876 | 0.14837465 | 0.11264702 | 0.15899703 | 1.41146241 | 0.2752 |
| **PA34:2** | 0.00724154 | 0.00657923 | 0.0064628 | 0.0105019 | 0.0070811 | 0.01124221 | 0.00676119 | 0.00960841 | 1.42111205 | 0.1266 |
| **PS 36:2** | 0.18715821 | 0.20777202 | 0.1743243 | 0.29053497 | 0.29198989 | 0.22969906 | 0.18975151 | 0.27074131 | 1.42682029 | 0.0495 |
| **GM3 18:1/22:1** | 0.00016701 | 0.00029323 | 0.0001181 | 0.00029621 | 0.00031661 | 0.00021449 | 0.00019278 | 0.00027577 | 1.43051629 | 0.1266 |
| **PE36:2p** | 0.01798535 | 0.01623829 | 0.01010785 | 0.02752977 | 0.01603733 | 0.02002606 | 0.01477716 | 0.02119772 | 1.43449161 | 0.2752 |
| **PE36:1** | 0.06383319 | 0.067739 | 0.05647913 | 0.09733988 | 0.08071078 | 0.0923068 | 0.06268378 | 0.09011915 | 1.43767908 | 0.0495 |
| **PG32:1** | 0.00223501 | 0.00173211 | 0.00142369 | 0.00280277 | 0.00263717 | 0.00232711 | 0.00179694 | 0.00258902 | 1.44079427 | 0.0495 |
| **LPI16:0** | 0.00231698 | 0.00242256 | 0.0024507 | 0.00321376 | 0.00304743 | 0.00411554 | 0.00239674 | 0.00345891 | 1.44317072 | 0.0495 |
| **LPI** | 0.02067908 | 0.02736215 | 0.02021147 | 0.03545484 | 0.03034883 | 0.03278349 | 0.0227509 | 0.03286239 | 1.44444331 | 0.0495 |
| **PI** | 0.93857269 | 1.07317851 | 0.74951777 | 1.70011307 | 1.14636536 | 1.16653551 | 0.92042299 | 1.33767131 | 1.45332236 | 0.0495 |
| **PE42:3p** | 0.01003481 | 0.01299761 | 0.01166621 | 0.01873742 | 0.01431434 | 0.01773768 | 0.01156621 | 0.01692981 | 1.46373048 | 0.0495 |
| **LPS** | 0.0324314 | 0.03966596 | 0.0326238 | 0.04836003 | 0.04453657 | 0.06045407 | 0.03490705 | 0.05111689 | 1.4643714 | 0.0495 |
| **GM3 18:0/16:0** | 0.00033346 | 0.00055819 | 0.00020288 | 0.00068918 | 0.00049133 | 0.00042757 | 0.00036485 | 0.00053603 | 1.46918733 | 0.2752 |
| **PI 38:5** | 0.09798749 | 0.11614636 | 0.07304452 | 0.18290292 | 0.11035285 | 0.12926033 | 0.09572612 | 0.1408387 | 1.47126713 | 0.1266 |
| **LPE18:0** | 0.04379478 | 0.03640181 | 0.02862398 | 0.06452244 | 0.04244441 | 0.05359835 | 0.03627352 | 0.05352173 | 1.47550419 | 0.1266 |
| **CE:16:0** | 0.21420106 | 0.21822721 | 0.21220634 | 0.33956055 | 0.22963181 | 0.38443947 | 0.21487821 | 0.31787728 | 1.47933698 | 0.0495 |
| **DAG40:6(18:0/22:6)** | 0.29229264 | 0.26188966 | 0.25344776 | 0.39910016 | 0.35686126 | 0.44307581 | 0.26921002 | 0.39967908 | 1.48463671 | 0.0495 |
| **PA36:2** | 0.01870611 | 0.02884337 | 0.01780627 | 0.03477414 | 0.02998644 | 0.0332257 | 0.02178525 | 0.03266209 | 1.49927558 | 0.0495 |
| **PC36:2** | 0.79328419 | 1.07294896 | 0.71209624 | 1.58707205 | 1.06891251 | 1.21310873 | 0.85944313 | 1.28969776 | 1.50062025 | 0.1266 |
| **LPE18:2** | 0.0307786 | 0.03350529 | 0.02053006 | 0.05093383 | 0.03688122 | 0.03976046 | 0.02827132 | 0.04252517 | 1.50418074 | 0.0495 |
| **PI 40:5** | 0.0080636 | 0.00798147 | 0.00703073 | 0.01603415 | 0.00984758 | 0.0088906 | 0.00769193 | 0.01159077 | 1.50687422 | 0.0495 |
| **PG** | 0.02458798 | 0.02661361 | 0.021098 | 0.03863817 | 0.03099168 | 0.03945091 | 0.02409986 | 0.03636025 | 1.50873266 | 0.0495 |
| **PG34:1** | 0.0090174 | 0.00954836 | 0.00805287 | 0.01584955 | 0.01112773 | 0.01320091 | 0.00887288 | 0.01339273 | 1.50940057 | 0.0495 |
| **LPA18:0** | 0.04341539 | 0.09073492 | 0.08233079 | 0.10496996 | 0.10157294 | 0.12030439 | 0.07216037 | 0.1089491 | 1.50981901 | 0.0495 |
| **PI 38:4** | 0.48553962 | 0.59461558 | 0.38863741 | 0.92205035 | 0.68001484 | 0.62929279 | 0.48959754 | 0.74378599 | 1.51917837 | 0.0495 |
| **LPI18:0** | 0.00995021 | 0.01421531 | 0.01041967 | 0.01892581 | 0.01656937 | 0.01762811 | 0.01152839 | 0.01770776 | 1.53601315 | 0.0495 |

| | | | | | | | | | | |
|---|---|---|---|---|---|---|---|---|---|---|
| **PA40:6** | 0.03771656 | 0.06295175 | 0.04428388 | 0.0892087 | 0.06667962 | 0.06677138 | 0.0483174 | 0.0742199 | 1.53609061 | 0.0495 |
| **PI 38:3** | 0.08533323 | 0.10164676 | 0.06115701 | 0.16467054 | 0.10831176 | 0.1097576 | 0.08271234 | 0.12757997 | 1.54245394 | 0.0495 |
| **PE32:1** | 0.01178012 | 0.01422704 | 0.00726126 | 0.02003871 | 0.01316942 | 0.01831256 | 0.01108947 | 0.01717356 | 1.54863641 | 0.1266 |
| **PE36:2** | 0.28220949 | 0.2712622 | 0.18679496 | 0.44700164 | 0.34290759 | 0.36404147 | 0.24675555 | 0.38465023 | 1.55883113 | 0.0495 |
| **LPS18:0** | 0.01932633 | 0.02599767 | 0.01988643 | 0.03125175 | 0.02809885 | 0.04262203 | 0.02173681 | 0.03399088 | 1.56374714 | 0.0495 |
| **PG34:2** | 0.00560016 | 0.00611141 | 0.00478036 | 0.00840407 | 0.00760118 | 0.00990163 | 0.00549731 | 0.00863563 | 1.57088212 | 0.0495 |
| **PA32:1** | 0.00105774 | 0.00162744 | 0.00108048 | 0.00230137 | 0.00158553 | 0.00212037 | 0.00125522 | 0.00200242 | 1.59527964 | 0.1266 |
| **PE40:5p** | 0.02563737 | 0.02537111 | 0.02189447 | 0.04801097 | 0.03511172 | 0.03376199 | 0.02430098 | 0.03896156 | 1.60329145 | 0.0495 |
| **PC36:5** | 0.01688487 | 0.01615809 | 0.01358923 | 0.03371576 | 0.02377929 | 0.0174577 | 0.01554406 | 0.02498425 | 1.60731791 | 0.0495 |
| **CE:18:3** | 0.02551849 | 0.04349288 | 0.02227577 | 0.05424284 | 0.04052326 | 0.0520054 | 0.03042905 | 0.04892383 | 1.60780051 | 0.1266 |
| **PG36:1** | 0.00190133 | 0.00173322 | 0.00161494 | 0.00283654 | 0.00222727 | 0.00339547 | 0.00174983 | 0.00281976 | 1.61144569 | 0.0495 |
| **PG36:4** | 0.00123309 | 0.00219043 | 0.00133827 | 0.00273099 | 0.00215855 | 0.00317436 | 0.00158726 | 0.00268796 | 1.69345761 | 0.1266 |
| **GM3 18:0/20:0** | 0.00023403 | 0.00020565 | 0.0001687 | 0.00044325 | 0.00028095 | 0.00042799 | 0.00020279 | 0.00038406 | 1.89389051 | 0.0495 |
| **PI 40:4** | 0.00502224 | 0.00839567 | 0.00622485 | 0.01724442 | 0.00949235 | 0.01113631 | 0.00654759 | 0.01262436 | 1.92809299 | 0.0495 |

Low Medium High

**lipid name abbreviations**

| | |
|---|---|
| **CE** | **cholesteryl ester** |
| **TAG** | **triacylglycerol** |
| **DAG** | **diacylglycerol** |
| **PC** | **phosphatidylcholine** |
| **PE** | **phosphatidylethanolamine** |
| **PA** | **phosphatidic acid** |
| **PS** | **phosphatidylserine** |
| **PI** | **phosphatidylinositol** |
| **PG** | **phosphatidylglycerol** |
| **LBPA** | **lyso-bisphosphatidic acid** |
| **LPA** | **lyso-PA** |
| **LPI** | **lyso-PI** |
| **LPS** | **lyso-PI** |
| **LPE** | **lyso-PE** |
| **GM3** | **monosialo-dihexosylganglioside** |

*P* values were caculated by two-tailed unpaired Student's *t*-test comparing lipid species betweeen WT and SA/SA groups.
$P < 0.05$ was considered as significant. The significant *P* values are highlighted in pink.

**Supplementary Table 2** Yeast strains used in this study.

| **Name** | **Parent** | **Genotype** | **Reference** |
|---|---|---|---|
| *wild-type* | BY4741 | *MATa his3Δ1 leu2Δ met15Δ ura3Δ gcn5Δ::kanMX6* | (30) |
| *gcn5Δ* | BY4741 | *MATa his3Δ1 leu2Δ met15Δ ura3Δ elp3Δ::kanMX6* | (30) |
| *elp3Δ* | BY4741 | *MATa his3Δ1 leu2Δ met15Δ ura3Δ hpa2Δ::kanMX6* | (30) |
| *sas2Δ* | BY4741 | *MATa his3Δ1 leu2Δ met15Δ ura3Δ sas2Δ::kanMX6* | (30) |
| *sas3Δ* | BY4741 | *MATa his3Δ1 leu2Δ met15Δ ura3Δ sas3Δ::kanMX6* | (30) |
| *rtt109Δ* | BY4741 | *MATa his3Δ1 leu2Δ met15Δ ura3Δ rtt109Δ::kanMX6* | (30) |
| *esa1-HA-aid* | BY4741 | *MATa his3Δ1 leu2Δ met15Δ ura3Δ ADH1-9XMYC-AtTIR1::HisMX6, esa1-HA-aid::URA3* | This study |
| *Wild-type-GFP-Faa4* | BY4741 | *MATa his3Δ1 leu2Δ met15Δ ura3Δ ADH1-9XMYC-AtTIR1::HisMX6, Faa4-GFP::KanMX6* | This study |
| *esa1-HA-aid-GFP-Faa4* | BY4741 | *MATa his3Δ1 leu2Δ met15Δ ura3Δ ADH1-9XMYC-AtTIR1::HisMX6, esa1-HA-aid::URA3, Faa4-GFP::KanMX6* | This study |
| *pah1Δ* | BY4741 | *MATa his3Δ1 leu2Δ met15Δ ura3Δ pah1Δ::URA3* | This study |
| *pah1Δ-vector* | BY4741 | *MATa his3Δ1 leu2Δ met15Δ ura3Δ pah1Δ::URA3 pRS316 [ vector-HIS3]* | This study |
| *pah1Δ-WT-PAH1* | BY4741 | *MATa his3Δ1 leu2Δ met15Δ ura3Δ pah1Δ::URA3 pRS316 [Myc-WT-PAH1 HIS3]* | This study |
| *pah1Δ-K496/K801R-PAH1* | BY4741 | *MATa his3Δ1 leu2Δ met15Δ ura3 Δpah1Δ::URA3 pRS316 [Myc-K496/K801R-PAH1 HIS3]* | This study |
